# Lobular Distribution and Variability in Hepatic ATP Binding Cassette Protein B1 (ABCB1, P-gp): Ontogenetic Differences and Potential for Toxicity

**DOI:** 10.3390/pharmaceutics9010008

**Published:** 2017-02-17

**Authors:** Ngu Njei Abanda, Zoe Riches, Abby C. Collier

**Affiliations:** 1 Department of Tropical Medicine, Medical Microbiology and Pharmacology, University of Hawaii, Honolulu, HI 96813, USA; abandann@hawaii.edu; 2 Biotechnology Center, University of Yaoundé I, Yaoundé, Cameroon; 3 Faculty of Pharmaceutical Sciences, University of British Columbia, 2405 Wesbrook Mall, Vancouver, BC V6T1Z3, Canada; zoe.riches@ubc.ca

**Keywords:** development, pediatric, systemic toxicity, elderly, obesity

## Abstract

The ATP Binding Cassette B1 (ABCB1) transporter has critical roles in endo- and xenobiotic efficacy and toxicity. To understand population variability in hepatic transport we determined ABCB1 mRNA and protein levels in total liver lysates sampled from 8 pre-defined sites (*n* = 24, 18–69 years), and in S9 from randomly acquired samples (*n* = 87, 7 days–87 years). ABCB1 levels did not differ significantly throughout individual livers and showed 4.4-fold protein variation between subjects. Neither mRNA nor protein levels varied with sex, ethnicity, obesity or triglycerides in lysates or S9 (that showed the same relationships), but protein levels were lower in pediatric S9 (*p* < 0.0001), with 76% of adult ABCB1 present at birth and predicted to mature in 5 years. Pediatric total liver lysates were not available. In summary, opportunistic collection for studying human hepatic ABCB1 is acceptable. Additionally, ABCB1 may be lower in children, indicating differential potential for toxicity and response to therapy in this special population.

## 1. Introduction

Interest in active transporters, such as the ATP Binding Cassette (ABC) proteins, has peaked in recent years because they play crucial roles in drug, chemical, hormone, and nutrient disposition [1]. Clinical and environmental studies increasingly consider the effects of transporters on therapeutic failure, drug resistance, and chemical toxicity. Despite this, very few published studies have determined transporter protein dynamics in human liver tissue samples—a critical site of transport action and critical mediator of systemic endo- and xenobiotic levels. Primarily, this is due to: (i) difficulties in obtaining human liver tissue samples; and (ii) technical challenges working with ABC proteins in the laboratory [2,3,4,5,6,7].

One of the important ABC transporters is ABCB1 (EC 3.6.3.44, P-glycoprotein, P-gp). The ABCB1 protein is present on the plasma membrane of almost all tissues of the human body (reviewed [8,9,10,11,12]). In the liver, it is expressed on the apical surface of hepatocytes. First characterized in the 1970s, ABCB1 is a 170 kDa protein composed of two sub-units, each containing six transmembrane domains, a large cytoplasmic domain, and a nucleotide binding domain [12]. Although ABCB1 actions in the liver are necessary for elimination of substances from the body to maintain homeostasis, excessive expression (such as by induction) can also reduce the therapeutic effects of drugs and upset homeostatic balance of e.g., hormones and bile acids. An example of this is the drug-drug interaction between Rifampicin (an ABCB1 inducer) and Apixaban, where the therapeutic effect of Apixaban is decreased through greater elimination, increasing risk of stroke [13]. Conversely low expression or activity of ABCB1 may increase the risk of toxicity by compromising elimination. This is observed in the Apixaban/Ketoconazole interaction (ABCB1 inhibition by the latter), where there is an increase in total Apixaban exposure and risk of haemorrhage [13]. 

We hypothesized that hepatic expression of ABCB1 would be greater in areas near the portal and bile circulation due to its physiological role as an efflux transporter. Currently, there are no published studies of differential regional expression of ABCB1 in the human liver. Regional differences in ABCB1 expression would have implications for sample collection and for differentiating between localized and systemic effects on drug and chemical disposition due to ABCB1 expression. In addition to understanding regional variability in hepatic ABCB1, we were interested in determining whether ABCB1 expression is associated with demographic parameters. Although studies associating expression with age, sex, and ethnicity have been common with metabolic enzymes [14,15,16,17,18,19,20,21,22] and have revealed critical insight into chemical action and toxicity, they largely do not exist for transport proteins. Based on similar roles as metabolic enzymes, we hypothesized that ABCB1 in the human liver would vary with age, sex, ethnicity, or obesity, and tested this in both the lysates as well as a larger cohort of 87 liver S9 fractions (7 days–87 years).

A greater knowledge of the spatial and demographic ABCB1 expression can help with understanding liver function and disease etiology, as well as systemic drug, chemical, and hormone effects mediated by liver transport.

## 2. Materials and Methods 

All chemicals and reagents were from Sigma-Aldrich (St. Louis, MO, USA) unless otherwise stated.

### 2.1. Tissue Samples

Liver S9 and lysate samples were released from the Hawaii Biorepository, with approval from the Institutional Review Board for Human Ethics at the University of Hawaii CHS15844 and the Review Ethics Board at the University of British Columbia H14-00092. The cohort was supplemented with samples purchased commercially from Cellzdirect (Durham, NC, USA), Puracyp (Carlsbad, CA, USA), and Xenotech (Lenexa, KS, USA). Table 1 summarizes donor demographics for the total liver lysates (*n* = 24). Table 2 summarizes the demographics of the S9 liver samples (*n* = 87). Lysates were prepared from approximately 5 g of frozen tissue. Tissues were thawed and weighed and homogenized 1:3 weight:volume in 0.1 M Tris-HCl containing 5 mM MgCl_2_ and 2 mM freshly prepared Phenylmethylsulfonyl fluoride. Tissues were homogenized to total lysate in 50 mL falcon tubes using a Tissue Tearor electrical homogenizer (Daigger Scientific, Vernon Hills, IL, USA). Total lysates were aliquoted and frozen at −80 until use.

### 2.2. Extraction of mRNA and cDNA Preparation

Total RNA (up to 5 µg) was extracted from human liver S9 (100 µL at 10–30 mg protein/mL). RNA (up to 6 µg) was also extracted from human liver lysates (50 µL at 4–50 mg protein/mL) using the RNeasy Mini kit according to the manufacturer’s instruction (Qiagen, Valencia, CA, USA). The RNA purity and concentration were determined by Nanodrop (ThermoFisher Scientific, Wilmington, DE, USA), then RNA was aliquoted stored at −80 °C until use. Only samples with 90% pure RNA or higher (OD260/280 > 0.8) were used for reverse-transcription with an ABI High Capacity Reverse Transcription Kit (Life Technologies, Burlington, ON, Canada).

### 2.3. SYBR Green q-RT-PCR and Primer Selection

Primer sequences for ABCB1 were (F): 5′-CAC CCG ACT TAC AGA TGA TG-3′ and (R) 5′-GTT GCC ATT GAC TGA AAG AA-3′ with predicted amplicon length of 81 and can be retrieved from NM_000927 [23]. The primers for 18S rRNA were (F): 5′-CAC GGC CGG TAC AGT GAA A-3′ and (R): 5′-AGA GGA GCG AGC GAC CAA-3′, with a predicted amplicon length of 71 and can be retrieved from NR_003286.2 [24].

Real-time PCR was performed on an ABI Step-one-plus real time PCR system (Life Technologies) using cDNA template (2.5 ng RNA equivalent from S9 samples or 5 ng RNA equivalent from total liver lysates). First, the forward and reverse primers concentrations were optimized to give best signal-to-noise amplification and were 300 nM for both forward and reverse primers for 18S and 300 nM forward and 500 nM reverse for ABCB1. Detection was with PerfeCTa SYBR Green SuperMix for IQ (Quanta BioSciences, Gaithersburg, MD, USA). Cycling conditions were: 1 cycle 30 s at 95 °C, 40 cycles of 5 s at 95 °C, 15 s at 60 °C, and 10 s at 70 °C then melt curve of 15 s at 95 °C, 60 s at 60 °C, and 15 s at 95 °C. The threshold value detection (*C*_T_) was set in the exponential phase of amplification and quantified by normalization to 18S rRNA. Analysis was performed on StepOne™ software version 2.3 (Applied Biosystems, Foster City, CA, USA), with *C*_T_ values converted to fold change differences using the 2^−ΔΔ*C*_T_^ method for relative quantitation [25].

### 2.4. Immunoblotting for Relative Expression of Protein

For western blotting of ABCB1, SDS-page gels (7%) were used to resolve 20 µg of liver lysate or S9 and each sample was analyzed on at least 3 separate gels as previously described [19]. Primary antibody was rabbit polyclonal anti-ABCB1 (ab129450, Abcam, Toronto, ON, Canada) incubated for 2 h at room temperature. Horseradish peroxidase conjugated donkey-anti-rabbit at 1:3000 was then incubated for 1 h at room temperature (Jackson Immunolabs, Westgrove, PA, USA). The membrane was developed for 1 min in enhanced chemiluminescence solution and detected on X-ray film for 60 min. Confirmation of even protein loading was by staining acrylamide gels with ponceau red and determining even loading. Additionally, a second variability control between blots run on different days was included. This second control was 20 µg of commercial human S9 from a pool of 200 individual livers, that was added to the left lane of every blot, and used to determine variability and to normalize expression. The inter-blot coefficient of variation (CV) of the XT200 was 14.6%, *n* = 9. Samples were semi-quantified with Image J 1.48v (http://imagej.nih.gov/ij) with background subtraction.

### 2.5. Total Triglyceride Liver Concentrations

The triglyceride colorimetric assay kit (Cayman Chemical Company, Ann Arbor, MI, USA) was used to determine the levels of triglycerides (mg/g of liver) as per manufacturer’s instructions. 

### 2.6. Statistical Analyses

All data sets were analyzed for normality with D’Agostino-Pearson tests. For sex or ethnicity (binary tests), student’s *t*-tests (two-tailed) were performed between groups. For binned continuous data (age, BMI), one way ANOVA with Tukey’s post hoc analysis was performed. Pediatric samples (<18 years) were not included in the BMI analysis, as BMI is not an appropriate measure for obesity in children. Where body weight was known, appropriate categories were assigned using the National Center for Health Statistics weight-for-age growth charts for children: underweight, <5th percentile; ideal weight, 5–85th; overweight, 86–94th; obese, >95th percentile (http://www.cdc.gov/growthcharts [26], Table 2). Correlations between mRNA and protein were performed using Pearson’s or Spearman’s correlation according to the normality distribution. A one-phase association (not forced through zero), straight line, and sigmoidal fits were compared to predict development of ABCB1 protein expression from birth. The best fit was determined using Aikake’s informative criteria (AIC), *F*-tests, sum of squares and residual analysis. All statistical analyses were performed using Prism 5.0 for Mac OsX (Graph Pad Prism, San Diego, CA, USA).

## 3. Results

### 3.1. Expression ABCB1 mRNA and Protein in Hepatic Lysates: Regional and Demographic Associations

**Figure 1:**
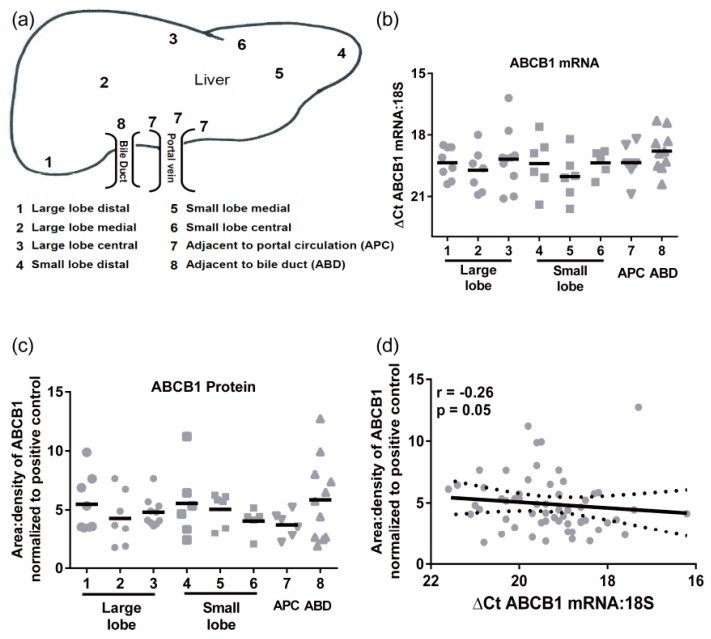

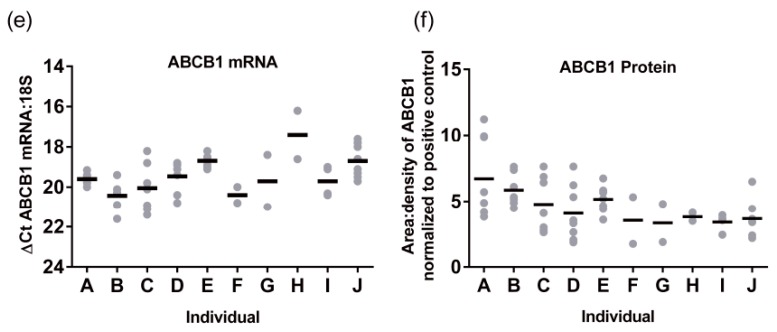
Expression of ABCB1 within different human liver regions. (**a**) Diagram of the different regions where samples taken within the same liver; (**b**) The mRNA expression of ABCB1 was determined within different regions of 14 different livers; (**c**) Protein expression of ABCB1 within different human liver regions: (distal large lobe *n* = 8; distal small lobe *n* = 6), medial large lobe *n* = 7; medial small lobe *n* = 6, central large lobe *n* = 10, central small lobe *n* = 5, APC = adjacent to portal circulation *n* = 6 and ABD = adjacent to bile duct N = 11); (**d**) Correlation between mRNA expression and protein expression for ABCB1, with 95 % confidence intervals (dotted line), and data analyzed by Spearman’s correlation; (**e**) ABCB1 mRNA expression (line = means) within the livers of 10 individuals; (**f**) The ABCB1 protein expression showing intra-individual variability of protein expression (8 regions for A, D, E, J; 7 regions for B and C; 4 regions for I and 2 regions for F, G and H).

Transporter mRNA expression was determined in a cohort of 24 individual livers using multiple pieces from each liver obtained from 8 distinct regions (not all samples were available from all individuals, Figure 1a). There were no significant differences in ABCB1 mRNA regional expression (Figure 1b). The mean *C*_T_ values (±SD) for 18S and ABCB1 were 13.3 ± 2.2 and 32.6 ± 2.8, respectively. The ABCB1 mRNA levels had an average 30-fold variability in mRNA levels. Similarly, there were no significant differences in protein expression of ABCB1 throughout the liver (Figure 1c). The highest level of protein and greatest range of ABCB1 expression was detected in the samples taken adjacent to bile ducts. The levels of ABCB1 mRNA and protein did not correlate (Figure 1d), indicating that ABCB1 expression is not purely transcriptional.

**Figure 2:**
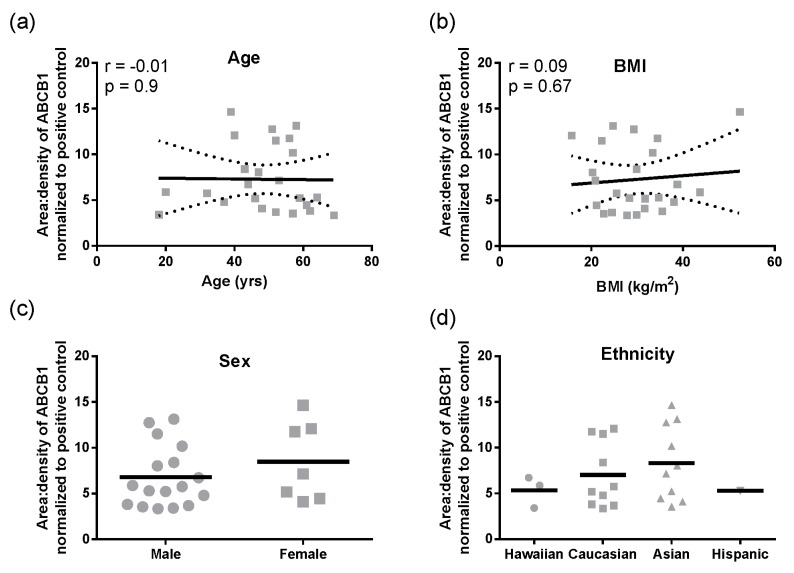
The protein expression of ABCB1 in a cohort of 24 liver lysates. The ABCB1 protein was detected by Western blotting and normalized to the ABCB1 levels detected in a pooled S9 liver sample (Xenotech, *n* = 200 individuals); (**a**) correlation between age and ABCB1 protein expression with dotted lines 95% confidence intervals; (**b**) Expression of ABCB1 protein compared with BMI; (**c**) The expression of ABCB1 proteins compared to sex; (**d**) Differences between ethnicity and ABCB1 protein expression.

Hepatic ABCB1 protein levels within the regional expression lysate cohort ranged from 3.4 ± 0.7 to 6.7 ± 3.0 area:density units (mean ± SD, Figure 1f). Lysates average five times more ABCB1 protein than S9 fractions. Intra-individual variability in hepatic ABCB1 protein expression, taken from eight different sites in the same liver, ranged from 1.2- to 4-fold with a mean value of 2.5 ± 0.9-fold (Figure 1f). In these total liver lysates, there was no significant correlation of ABCB1 protein with age, BMI, sex, or ethnicity (Figure 2a–d). However, for ethnicity it should be noted that statistical comparisons essentially compare Caucasians and Asians due to the small sample size for Hispanics and Hawaiians.

**Figure 3:**
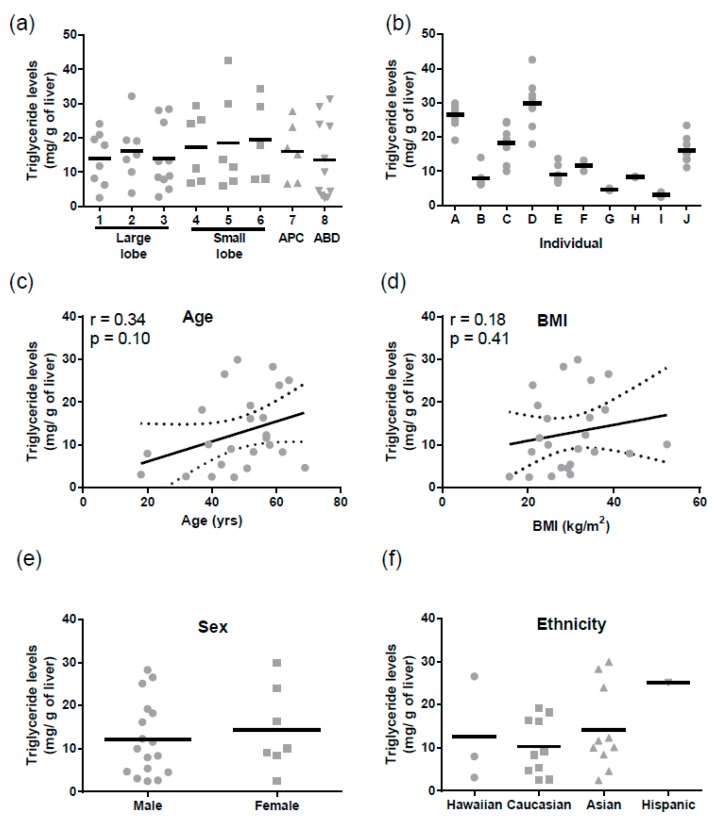
Triglyceride levels in human liver lysates. (**a**) The regional expression of hepatic triglyceride levels: 1 & 4 distal, 2 & 5 medial, 3 & 6 central, APC = adjacent to portal circulation and ABD = adjacent to bile duct, see Figure 1a for detailed liver regions; (**b**) The inter- and intra-individual expression of triglycerides levels. The correlation of triglyceride levels with (**c**) age and (**d**) BMI. The relationship between triglyceride levels and (**e**) sex and (**f**) ethnicity.

Similarly, for triglycerides mean levels were 15.4 ± 1.2 mg/g of liver (mean ± SD). There were no regional differences in triglyceride levels (Figure 3a), but triglycerides varied significantly between individuals (Tukey *p* < 0.0001, Figure 3b). There was no correlation between hepatic triglyceride levels and age, BMI, sex, or ethnicity (Figure 3c–f). Triglyceride levels did not correlate with ABCB1 mRNA (r = −0.004, *p* = 0.98) or protein (r = −0.23, *p* = 0.30).

### 3.2. Expression of ABCB1 in Hepatic S9: Demographic and Ontogenetic Associations

**Figure 4:**
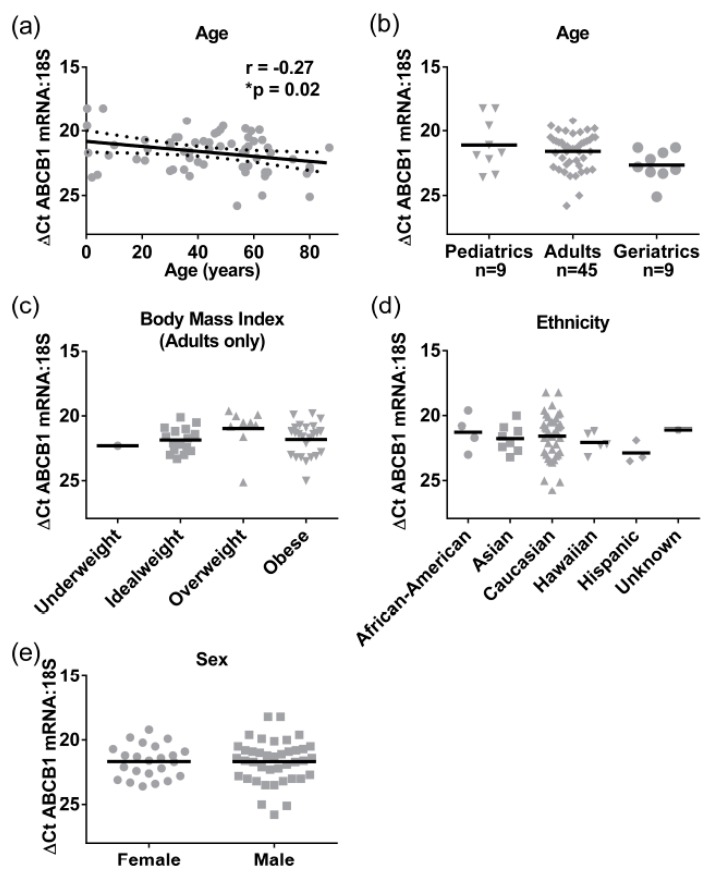
The mRNA expression of ABCB1 in a cohort of 80 liver samples. Gene expression was measured using SYBRGreen detection and ABCB1 gene expression was normalized to 18S to give Δ*C*_T_ value. (**a**) Line graph shows linear regression with 95% confidence intervals, (dotted line), of age compared to gene expression. Data analyzed by Pearson’s correlation (* *p* < 0.05); (**b**) Dot blot compare gene expression levels with age grouped into pediatric (≤18 years), adult (19–64 years), and geriatric (≥65 years) and data showing mean (horizontal line). The mRNA expression was also compared to (**c**) obesity, measured by BMI, (**d**) Ethnicity, and (**e**) Sex.

Because we only had adult total liver lysates, to provide further insight into the ontogeny of ABCB1 we analyzed a cohort of liver S9 where pediatric, adult, and elderly samples were available (Table 2). The ABCB1 mRNA (*n* = 79) was detectable from birth, and a significant negative correlation with age was observed (Pearson r = −0.3, *p* = 0.02), although the correlation coefficient suggested that this was only a moderate association, with 8% of the change in mRNA expression being attributed to age as a covariate (Figure 4a). When grouped into categories, the elderly (>65 years, mean Δ*C*_T_ ± SEM of 22.7 ± 0.4) had lower ABCB1 mRNA levels than adults (mean Δ*C*_T_ ± SEM of 21.6 ± 0.2, Figure 4b). Although not statistically significant, this 2-fold difference in gene expression is driving the negative association. Exactly the same as in the case of the total lysates, there were no significant differences observed for BMI, ethnicity, or sex in ABCB1 mRNA expression levels in S9 (Figure 4c–e). Pediatric samples (<18 years) were not included in the BMI analysis, as BMI is not an appropriate measure for obesity in children; rather, percentile-weight-for-age (National Center for Health Statistics) was used.

**Figure 5:**
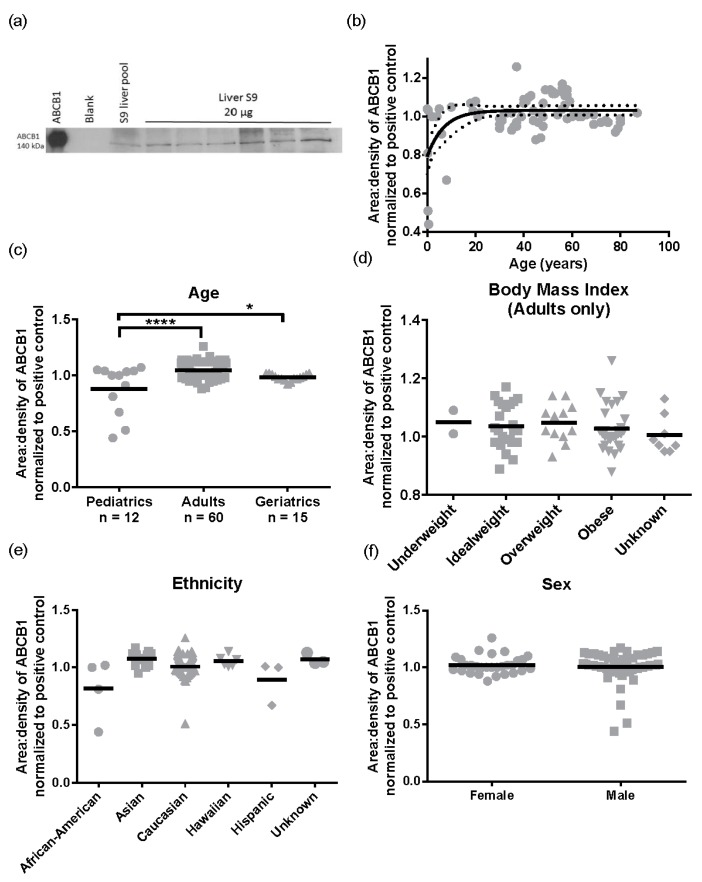
Protein expression of ABCB1 in a liver cohort. The ABCB1 protein was detected by Western blotting and normalized to the ABCB1 levels detected in a pooled S9 liver sample (Xenotech, *n* = 200 individuals). (**a**) Example of Western blot of 6 individuals with 20 µg liver S9 loaded compared with pooled S9 sample, ABCB1 = recombinant ABCB1 expressed in baculosome (5 µg), and blank = baculosome with no expression (5 µg); (**b**) Relationship between age and ABCB1 protein expression with a one-phase association, with 95% confidence intervals (dotted line); (**c**) Protein expression compared to individuals grouped by age and analyzed by ANOVA (*p* < 0.0001) and ABCB1 expression in pediatrics was significantly lower compared to adults and geriatrics (Tukey’s multiple comparison test, *** *p* < 0.0001, * *p* < 0.05); (**d**) Expression of ABCB1 protein in obese individuals, measured by BMI; (**e**) Differences between ethnicity and ABCB1 protein expression; (**f**) The expression of ABCB1 proteins compared to sex.

Subsequently, we performed the same analysis for ABCB1 protein levels (*n* = 87). A representative Western blot image for ABCB1 detection is presented in Figure 5a. There was a 2.9 ± 0.32 -fold variability for ABCB1 protein levels in the population sampled ranging from 0.44 to 1.26 area:density units. Again, the same relationships were observed in S9 as total liver lysates, with no significant differences in ABCB1 protein expression observed for obesity (BMI, range 15.8–57.6) ethnicity, or sex (Figure 5d–f). However, different to total lysates where no pediatric samples were available, children have lower ABCB1 than adults. The ABCB1 protein was detected from birth and increased with age following a mono-exponential rise-to-plateau relationship with best fit (*F*-test; and fit parameters of AICc, 15.56, Sum of Squares 0.77, Figure 5b). This model predicts protein levels are at 76% of adult levels at birth and reach adult levels (±10%) by 5 years of age. Using categorical data, children had a significantly lower ABCB1 protein levels than adults (*p* < 0.001, Figure 5c). Although the elderly population had a trend towards lower ABCB1 expression than adults, this was not significantly different. 

## 4. Discussion

One of the key findings in this study was that ABCB1 protein expression does not differ significantly in different liver regions; this indicates that random collection of liver tissue is appropriate for studying ABCB1 ex vivo. The caveat to this finding is that in standardizing our assays on a per milligram of protein basis, we have assumed that there are equal numbers of hepatocytes per milligram of tissue in each of the 8 liver regions sampled. Although tissues did not differ visually in any way, but we did not confirm cell types histologically. No significant differences were associated with ethnicity, sex, or obesity (measured by both BMI and liver fat levels). 

The BMI parameter is frequently used as a measure for obesity; however, this can be flawed [27,28], and many healthy individuals have high BMI but would have low internal fats (for example certain athletes) [29]. We therefore additionally evaluated liver triglyceride levels to understand if the pathological manifestation of obesity (liver fat) could alter ABCB1 expression. Although the ABCB1 transporter is known to be involved in endogenous movement of cholesterol, phospholipids, and sphingolipids, and therefore plays a role in lipid homeostasis [30], neither triglyceride levels nor BMI were related to differences in ABCB1, a novel finding.

Finally, because we did not have pediatric or significant numbers of elderly liver lysates, we used S9 to determine ontogenetic differences in ABCB1. Children (≤18) have significantly lower and more variable expression of hepatic ABCB1 protein than adults, with ~76% of adult ABCB1 protein levels present at birth, reaching full maturity by five years of age. The strength of the data presented is that in every other category tested, S9 results were reflective of liver lysates, albeit with lower absolute levels of mRNA and protein present. In addition, all of the S9 samples were treated the same way i.e., similarly depleted of membranes, so the relative variability of the ABCB1 proteins should be preserved, even though absolute levels are not. 

Relatively few studies of pediatric ABCB1 and ontogeny exist. Miki et al., 2005, reported no differences in hepatic ABCB1 mRNA but significant decreases in mRNA expression in the lungs of the elderly [4]. Moreover, several studies have investigated the ontogeny of intestinal ABCB1 in humans [5,31,32,33]. Immunohistochemistry techniques have demonstrated that the transporter is differentially expressed in children under the age of three: ABCB1 is present on both the apical and basolateral surfaces of enterocytes, while after three the protein is only detected apically [31]. Recently, lower neonatal intestine mRNA levels have been reported that reach adult levels in early childhood [5]. However, subsequent tests of hepatic and intestinal proteins by the same authors using LCMS showed stable expression of ABCB1 from fetus to adult, albeit in fewer samples than presented here [33]. These studies suggest that organ-specific and ontogenetic regulation of ABCB1 protein is likely, albeit with some disagreement in the literature. 

## 5. Conclusions

In summary, investigators who collect human liver samples opportunistically, as well as those who collect uniformly from a single site, likely have a cohort representative of population variability in ABCB1 that can be accurate for extrapolation. Additionally, children under five express less hepatic ABCB1 than the general population, which could cause xeno- and endobiotic toxicity including altered responses to therapy, but this requires confirmation in a larger study, preferably using total liver lysates. Better understanding of the natural expression patterns of ABCB1 in the human liver can assist in translating work from human tissues into understanding of the mechanisms of drug and chemical efficacy, toxicity, and resistance.

## Figures and Tables

**Table 1:** Demographic information of the donors used to investigate regional ATP Binding Cassette B1 (ABCB1) expression in the liver.

ID	Number of Liver Samples Taken for Regional Study	Age (Years)	BMI (Kg/m^2^)	Sex	Ethnicity
A	8	44	38.8	Male	Hawaiian or other pacific Islander
B	7	20	43.7	Male	Hawaiian or other pacific Islander
C	7	37	38.0	Male	Caucasian
D	8	48	31.6	Female	Asian
E	8	46	31.7	Female	Caucasian
F	2	57	22.7	Male	Asian
G	2	69	27.8	Male	Caucasian
H	2	62	35.5	Male	Caucasian
I	4	18	29.9	Male	Hawaiian or other pacific Islander
J	8	52	24.5	Male	Caucasian
K	1	61	21.1	Female	Japanese
L	1	51	29.3	Male	Asian
M	1	47	20.3	Male	Asian
N	1	32	25.5	Male	Caucasian
O	1	57	33.4	Male	Asian
P	1	58	24.7	Male	Asian
Q	1	40	15.7	Female	Caucasian
R	1	43	29.9	Male	Caucasian
S	1	59	28.3	Male	Asian
T	1	53	20.9	Female	Asian
U	1	52	22.3	Male	Caucasian
V	1	56	34.4	Female	Caucasian
W	1	39	52.4	Female	Asian
X	1	64	34.7	Male	Hispanic

**Table 2:** Demographic information of the liver cohort.

Group Age Range	Age Mean ± SD (Years) *n*	Ethnicity		Sex		Body Mass Index (BMI)	
Population 0.018–87 years	44.8 ±22.5 *n* = 87	Caucasian Asian Pacific Islander African American Hispanic Other/unknown	68% 14% 7% 5% 3% 3%	Female Male Unknown	33% 66% 1%	Underweight (BMI ≤ 18.9) Ideal weight (19–24.9) Overweight (25–29.9) Obese (30–39.9) Morbidly obese (≥40.1) Unknown/not included	3% 31% 16% 26% 9% 14 %
Pediatrics 7 days—18 years	4.6 ±5.4 *n* = 12	African-American Caucasian Pacific Islander Hispanic Other/unknown	*n* = 2 *n* = 6 *n* = 1 *n* = 1 *n* = 2	Female Male Unknown	*n* = 2 *n* = 9 *n* = 1	Underweight (<5th percent) Ideal weight (6–85th percent) Overweight (86–94th percent) Obese (>95th percent) Unknown/not included	*n* = 1 *n* = 3 *n* = 2 *n* = 2 *n* = 4
Adult 19—64 years	45.3 ±12.9 *n* = 60	Caucasian Asian Pacific Islander African American Hispanic Other/unknown	*n* = 39 *n* = 12 *n* = 4 *n* = 2 *n* = 2 *n* = 1	Female Male	*n* = 20 *n* = 40	Underweight (BMI ≤ 18.9) Ideal weight (19–24.9) Overweight (25–29.9) Obese (30–39.9) Morbidly obese (≥40.1) Unknown/not included	*n* = 2 *n* = 19 *n* = 10 *n* = 19 *n* = 5 *n* = 5
Geriatrics 65–87 years	74.6 ±6.7 *n* = 15	Caucasian Pacific Islander	*n* = 14 *n* = 1	Female Male	*n* = 7 *n* = 8	Underweight (BMI ≤ 18.9) Ideal weight (19–24.9) Overweight (25–29.9) Obese (30–39.9) Morbidly obese (≥40.1) Unknown/not included	*n* = 0 *n* = 5 *n* = 2 *n* = 2 *n* = 3 *n* = 3
